# Not a cakewalk: Insights into movement of large carnivores in human‐dominated landscapes in India

**DOI:** 10.1002/ece3.7156

**Published:** 2021-01-22

**Authors:** Bilal Habib, Pallavi Ghaskadbi, Shaheer Khan, Zehidul Hussain, Parag Nigam

**Affiliations:** ^1^ Wildlife Institute of India Chandrabani India

**Keywords:** canids, core areas, displacement, felids, movement ecology, radio telemetry

## Abstract

Large carnivores play an important role in the functioning of ecosystems, yet their conservation remains a massive challenge across the world. Owing to wide‐ranging habits, they encounter various anthropogenic pressures, affecting their movement in different landscape. Therefore, studying how large carnivores adapt their movement to dynamic landscape conditions is vital for management and conservation policy.

A total of 26 individuals across 4 species of large carnivores of different sex and age classes (*14 Panthera tigris, 3 Panthera pardus, 5 Cuon alpinus, and 4 Canis lupus pallipes*) were GPS collared and monitored from 2014–19. We quantified movement parameters (step length and net squared displacement) of four large carnivores in and outside protected areas in India. We tested the effects of human pressures such as human density, road network, and landuse types on the movement of the species. We also examined the configuration of core areas as a strategy to subsist in a human‐dominated landscape using BBMM.

Mean displacement of large carnivores varied from 99.35 m/hr for leopards to 637.7 m/hr for wolves. Tigers outside PAs exhibited higher displacement than tigers inside PAs. Moreover, displacement during day–night was significantly different for tigers inside and outside PAs. Similarly, wolf also showed significant difference between day‐night movement. However, no difference in day–night movement was found for leopard and dholes. Anthropogenic factors such as road length and proportion of agriculture within the home range of tigers outside PAs were found to be significantly different. All the habitat variables in the home range showed significant difference between the social canids. The core area size for tiger outside PA and wolf was found greater than PAs.

The study on movement of large carnivore species across landscapes is crucial for conservation planning. Our findings can be a starting point for interlinking animal movement and landscape management of large carnivore conservation in the current Anthropocene.

## Graphical abstract

The study is about movement parameters of four large carnivores in and outside protected areas in India (tiger, leopard, dhole, and wolf). In this study, we tested the effects of human pressures like human density, road density, and landuse types on the movement of the species. Finally, we examined the configuration of core areas as a strategy to exploit the human‐dominated landscape.

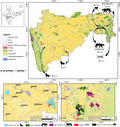

## 1 INTRODUCTION

Across the globe, large carnivores are considered as the most charismatic yet vulnerable components of their ecosystems (Miquelle et al., 2005). Positioned at the top of food chains, they influence all trophic levels, thereby shaping the entire community (Ripple et al., 2014). However, throughout their distributional range, large carnivore populations continue to decline rapidly due to anthropogenic pressures such as habitat degradation and fragmentation, depletion of wild prey, persecution, and illicit commercial trade in body parts (Weber & Rabinowitz, 1996).

Owing to their wide range requirements, large carnivores inherently occur at low densities across their distribution (Woodroffe & Ginsberg, 1998). However, the idyllic contiguous landscapes required for the long‐term conservation of such species are being increasingly compromised due to competition with humans over space. To survive, large terrestrial predators must negotiate human‐modified landscapes adjoining protected areas (PAs) which are under various landuse types. Such peculiar scenarios may lead to perceived or potential human–wildlife conflict posing a risk to the existence of wildlife in the area. Consequently, large carnivore conservation has become the prime focus of various stakeholders from scientists to policymakers (Linnell et al., 2001; Treves, 2009; Weber & Rabinowitz, 1996).

India is known for its rich biodiversity and is home to the highest number of large terrestrial carnivores (average body weight > 15 kg) in the world (Johnsingh, 1986). It also ranks 2nd in the world human population with 1.3 billion people and a density of 450 people per km^2^ (UN World Population Report, 2017). Based on the World Bank Report (2015), 60.4% of the total land in India is under agriculture resulting in a habitat matrix of human agricultural landscapes interspersed with PAs. As a result, humans are in direct competition with wildlife over limited resources, particularly, space. India is also home to 25% of world's cattle and holds the highest number of the world's livestock (19th All India Livestock Census, 2012). In conjunction with agriculture, the country's total road length is spread over 5.6 million km, with the highest global density of 1.70 km roads per square kilometer of land (Basic Road Statistics of India, 2016).

In this setting, survival of large carnivores depends on their ability to adapt to the human‐modified environment. The movement parameters of species are shaped in response to the dynamic structure of a landscape (Fahrig, 2007) and plays a major role in obtaining resources, evading threats, dispersing and finding mates (Clobert et al., 2009; Swingland & Greenwood, 1983). Consequently, this affects population dynamics through genetic connectivity as well as individual fitness (Morales et al., 2010; Nathan et al., 2008). Extrinsic factors such as habitat quality, resource availability, as well as anthropogenic features (settlement, roads, landuse changes, population density) also influence animal movement. Many studies have shown that anthropogenic features may affect animal movement either way (Andersen et al., 2017; Evans et al., 2019; Kerley et al., 2002; Kozakai et. al., 2013; Trombulak & Frissell, 2000; Webb et al., 2011).

Large carnivores exhibit different movement patterns and space use across landscapes due to their wide‐ranging and varied territorial behavior. The rapid rate at which landscapes are changing may compel wide‐ranging terrestrial mammals to adapt and change their movement patterns for long‐term survival. The PAs in India are small, isolated with compromised functional connectivity (Chundawat et al., 2016; Mondal et al., 2016) and wide‐ranging large carnivores need to move through areas with varying degrees of human activity to maintain healthy populations. However, they may be reluctant to cross certain habitat boundaries (Haddad, 1999). The study of movement parameters of such species is imperative to gain insights into fundamental biological processes like dispersal strategies, foraging, social interactions, and general patterns of space use that play a major role in determining community and population structures (Nathan et al., 2008).

The advancement of GPS technology has revolutionized animal tracking studies (Cagnacci et al., 2010; Kays et al., 2015). The fine‐scale location data at varied temporal and spatial scales allow more rigor and accuracy in such studies. In this paper, we studied the movement parameters of four large carnivores in the Central Indian Landscape, India. We evaluated the movement patterns of tiger (*Panthera tigris)*, leopard (*Panthera pardus)*, dhole (*Cuon alpinus)*, and wolves *(Canis lupus pallipes)* in different systems, that is, protected area and outside protected area. We examined the effect of landuse, human density, and road length as surrogates of human footprint on the movement of these wide‐ranging species across PAs and outside PAs. We hypothesized that 1. species outside PA would travel more (i.e., with longer displacement) than present in PA, 2. species will move faster at night in outside PA, and 3. species movement will be more in the human‐dominated landscape because of environmental and anthropogenic factors.

## 2 MATERIALS AND METHODS

### 2.1 Study area

**FIGURE 1:**
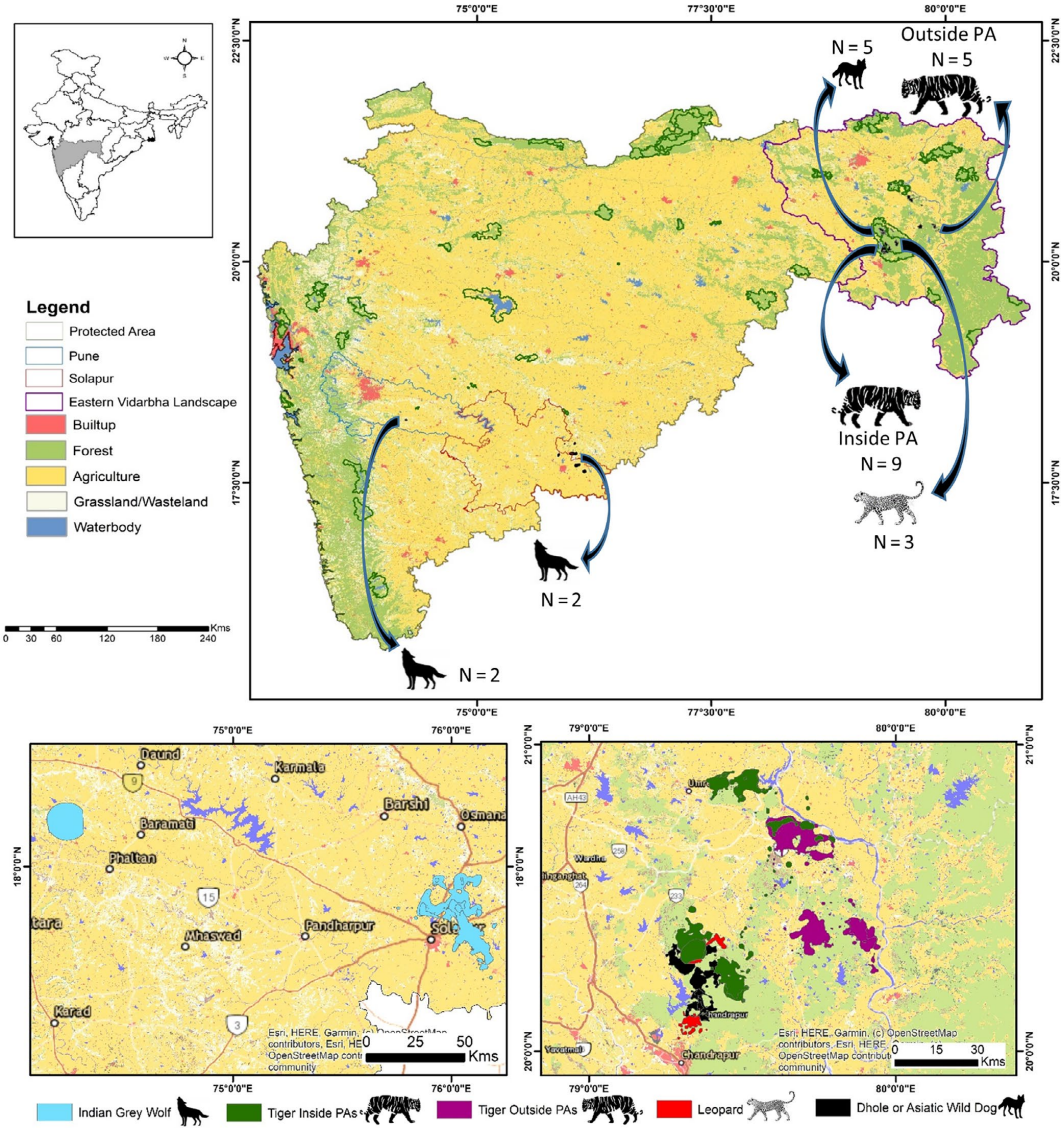
Map of study sites (top) with landuse and protected areas, (below left) home ranges of wolves and (below right) home ranges of tigers inside and outside PA, leopard, and dhole in Maharashtra, India

The study was conducted across various PAs and outside PAs in the state of Maharashtra, India. This includes the Eastern Vidarbha Landscape (EVL) of the Nagpur and Chandrapur Divisions and districts of Pune and Solapur. The study on tigers, dholes, and leopards was conducted in EVL across 2 PAs (Tadoba Andhari Tiger Reserve and Umred Karhandla Wildlife Sanctuary) and outside PA (Brahmapuri Forest Division). EVL encompasses an area of approximately 50,000 km^2^ and 40% of forest cover of the total area. It also has 8,540 villages with a human population of >10 million people which makes the landscape matrix of agricultural lands and wildlife areas. (Habib et al., 2017). The habitat in the landscape is primarily tropical dry deciduous forest with teak (*Tectona grandis)* and bamboo *(Dendrocalamus strictus)* as the dominant flora and is home to an estimated number of 312 tigers (range 270–354) (Jhala et al., 2020). The study on wolves was conducted across the grasslands of semi‐arid landscapes in two districts of Pune and Solapur in Maharashtra. This semi‐arid region receives less rainfall that makes it suitable for wolves. The summer season is very dry and extremely hot, with temperatures regularly exceeding 45°C. The terrain is gently undulating with mild slopes and flat‐topped hillocks with intermittent shallow valleys, which form the major drainage channels. Crop fields, grazing lands, scrublands, grasslands, villages, and open forest (Figure 1) dominate the area.

### 2.2 Study species

The Tiger (*Panthera tigris tigris*), Asia's largest obligate terrestrial carnivore is categorized as Endangered under the IUCN Red List of Threatened Species. In India, it is listed in Schedule I of the Indian Wildlife (Protection) Act, 1972, under the highest level of protection. Tigers are wide‐ranging, territorial felids, and Tropical Dry Forest is the largest habitat that supports tiger populations in the Indian subcontinent (Smith et al., 2011; Wikramanayake et al., 1998). Most of the tiger populations are present in PA’s but their size in India is too small to maintain viable populations of this species over time. Several studies on tigers were carried out to understand the home ranges patterns and size of home range can be highly variable across their habitat and landscape (Chundawat et al., 1999; Goodrich et al., 2010; Jhala et al., 2010; Naha et al., 2016; Sarkar et al., 2016; Sunquist, 1981). However, information on their movement parameters and the impact of environmental and anthropogenic features is not studied so far in India.

The leopard *(Panthera pardus)* is a highly adaptable, widely distributed felid, and is listed as Vulnerable under the IUCN Red List. In India, the leopard is also listed in Schedule I of the Indian Wildlife (Protection) Act, 1972. Wherever leopards coexist with tigers, lions, and dholes, a high degree of intraguild competition is observed (Hayward & Slotow, 2009; Wang & Macdonald, 2009). Leopards display great behavioral plasticity by shifting feeding preferences, space use, microhabitat use, and activity pattern (Karanth & Sunquist, 2000) which enables them to survive in human‐altered landscapes.

The Asiatic wild dog *(Cuon alpinus)* or dhole, is a social canid and is the only extant species of the genus *Cuon*. The monotypic species is listed under the Endangered category of the IUCN Red List and is protected under Schedule II of India's Wildlife (Protection) Act, 1972. Throughout their range, dholes are one of the top predators of tropical forests. In India, dholes share habitat with large carnivores like the tiger and leopard. Previous studies on dholes have focused on the intraguild competition, behavioral ecology, and genetics (Acharya, 2007; Ghaskadbi et al., 2016; Habib, Ghaskadbi, et al., 2018; Hayward et al., 2014; Johnsingh, 1980; Modi et al., 2018) but information on their movement ecology is limited.

The Indian wolf *(Canis lupus pallipes)* is distributed across Central India, up to Rajasthan in the north and Karnataka in the south (Shahi, 1982), and their population is estimated at 2000–3000 individuals (Jhala, 2000). They are categorized as Endangered by the IUCN Red List of Endangered Species. It is protected under Schedule I of the Wildlife (Protection) Act 1972. The Indian wolf is an iconic top predator in the open grasslands and adapted themselves to survive in the human‐dominated landscape (Shahi, 1982; Jhala, 1991; Habib, 2007. Studies on *C*. *l*. *pallipes* suggest that this species is a part of an ancient clade which has not mixed with the wolf‐dog clade, making them unique among other wolves of the world (Sharma et al., 2004; Shrotriya et al., 2012). Few studies have been conducted to estimate home range size but information on their movement is not studied so far in India. The average home range reported using minimum convex polygon method for three packs of Indian wolf ranged from 113.4 to 227.6 km^2^ (Jethva, 2003). The study conducted in southern Maharashtra found the average home range of the four packs was 183.58 ± 22.9 km^2^, with the average core area (50% MCP) of 9.74 km^2^ (Habib, 2007).

### 2.3 Capture and radio‐collaring

Overall, 26 individuals across 4 species of large carnivores were radio‐collared (Figure 1) and monitored from years 2014–19. The animals were fitted with GPS collars that were programmed to take fixes at different intervals (Table 1). The GPS data was downloaded from satellite links (Iridium and Globalstar) as well as UHF ground download receiver. The animals were intensively tracked in the field using VHF ground tracking.

**TABLE 1:** Species‐wise detail of each individual's characteristics, number of locations used, habitats, and type of collars used to study the movement of 4 large carnivores in India

Species	Individual ID	Sex	Age	Habitat/System	GPS location acquired	Monitoring days	Monitoring period	Collar type
Wolf	W1	Female	Subadult	Outside PA	6,748	615	25.12.17 to 01.09.19	Iridium, UHF/VHF/Activity
Wolf	W2	Male	Subadult	Outside PA	2,148	217	28.12.17 to 01.08.18	Iridium, UHF/VHF/Activity
Wolf	W3	Female	Adult	Outside PA	6,049	604	22.01.18 to 16.09.19	Iridium, UHF/VHF/Activity
Wolf	W4	Male	Adult	Outside PA	VHF Collar	604	22.01.18 to 16.09.19	VHF/Proximity Collar
Tiger	T07	Female	Adult	PA	1,871	520	17.10.14 to 20.03.16	Iridium, VHF/Activity
Tiger	Umred F	Female	Subadult	PA	2,109	308	12.03.18 to 13.01.19	Iridium, VHF/Activity
Tiger	T17	Female	Subadult	PA	1,687	267	07.03.17 to 28.11.17	Iridium, VHF/Activity
Tiger	T42	Male	Adult	PA	1,301	166	19.10.14 to 02.04.15	Iridium, VHF/Activity
Tiger	T09	Male	Subadult	PA	837	148	18.03.16 to 12.08.16	Iridium, VHF/Activity
Tiger	T10	Male	Subadult	PA	712	113	18.03.16 to 08.07.16	Iridium, VHF/Activity
Tiger	T7‐C1	Male	Subadult	PA	3,324	358	10.06.18 to 02.06.19	Iridium, VHF/Activity
Tiger	T7‐C2	Male	Subadult	PA	1,532	183	09.06.18 to 08.12.19	Iridium, VHF/Activity
Tiger	T103	Male	Subadult	PA	2,135	375	09.03.18 to 18.03.18	Iridium, VHF/Activity
Tiger	T01	Male	Adult	Outside PA	1,097	217	15.09.15 to 19.04.16	Iridium, VHF/Activity
Tiger	T9 brh	Male	Subadult	Outside PA	4,870	549	12.08.16 to 17.02.18	Iridium, VHF/Activity
Tiger	T10 brh	Male	Subadult	Outside PA	2,440	284	09.07.16 to 18.04.17	Iridium, VHF/Activity
Tiger	E3	Female	Subadult	Outside PA	3,747	329	02.01.19 to 26.11.19	Iridium, VHF/Activity
Tiger	Brh M	Male	Subadult	Outside PA	833	155	03.06.16 to 04.11.16	Iridium, VHF/Activity
Leopard	L25	Female	Adult	PA	48	38	03.08–13 to 09.09.13	GPS Global Star/VHF
Leopard	L26	Female	Adult	PA	297	462	03.08.13 to 07.11.14	GPS Global Star/VHF
Leopard	L41	Male	Adult	PA	96	415	23.04.15 to 10.06.16	GPS Global Star/VHF
Dhole	D1	Male	Adult	PA	1,799	77	29.07.17 to 13.10.17	GPS Plus UHF 1C Activity/VHF
Dhole	D2	Male	Adult	PA	1,407	177	25.10.17 to 19.04.18	GPS Plus UHF 1C Activity/VHF
Dhole	D3	Female	Adult	PA	1,007	58	20.02.18 to 18.04.18	GPS Plus UHF 1C Activity/VHF
Dhole	D4	Male	Subadult	PA	441	20	14.02.18 to 05.03.18	GPS Plus UHF 1C Activity/VHF
Dhole	D5	Male	Adult	PA	111	16	24.05.18 to 08.06.18	GPS Plus UHF 1C Activity/VHF

We captured 14 tigers (nine from PAs; five outside PA) across different age and sex classes (Table 1). The captured tigers were initially identified for collaring by field‐based monitoring and camera trapping. After identification, the individuals were tracked and immobilized using combination of Medetomine hydrochloride, Ketamine hydrochloride, and Xylazine (dosages based on the body weight, age, and sex). Dosage was injected remotely using an air‐pressurized Dan‐Inject projector (Model IM) from an open‐top vehicle, and the immobilized animal was approached. Collared tigers were monitored intensively between 2014–19 to study their movement and ranging patterns. We followed the same protocol for capturing dholes and used the drug combination of Tiletamine and Zolazepam (Zoletil 100, Virbac) (Van Heerden et al., 1991). The drug mixture was delivered from a vehicle remotely using a Dan‐Inject projector (Model JMSP.25). We captured 5 dholes across age and sex classes including three adult males, one subadult male, and one adult female. The dholes were intensively monitored from 2017–18 to study their ranging pattern. Furthermore, 3 leopards (two females and one male) were captured using baited cage and monitored from 2014–15. Baited cage was allured by a live goat (to lure the animal toward the trap) kept in a separate chamber inside the cage, and when the animal approaches the prey, a mechanical trapping system gets activated to slide down the rear door to trap the animal. The trapped animals were immobilized using a drug mixture of Ketamine and Xylazine. Between 2017 and 2018, 4 wolves consisting of two males and two females were collared in the semi‐arid landscape of Maharashtra. Wolves were captured using soft‐catch leghold traps. Traps (*n* = 25) were set up in a circle, placed ~20 cm away from each other, and wolf gland lure No. 100 (Stanley Hawbaker and Sons, Fort London, Pennsylvania) was used as an attractant to trap wolves (Habib, 2007). Traps were monitored continuously and trapped wolves were captured using double‐threaded nylon hockey net (Habib & Kumar, 2007) and immobilized using a Ketamine–Xylazine drug mixture. The average time for capturing of an individual wolf was 41.06 ± 21.54 hr.

### 2.4 Understanding movement parameters

We assessed the movement patterns of 4 large mammals using two movement parameters, such as mean displacement (step length) and net squared displacement (NSD). Displacement is defined as the straight‐line distance between two consecutive GPS locations of an animal trajectory. Varying interfix intervals across species were made uniform by postprocessing all data into an hourly data format for further analysis (Abrahms et al., 2017; Leblond et al., 2016). Mean displacement during day and night was also compared across individuals and landscapes by classifying location using animal movement tool (amt).

We also calculated NSD, which is the squared distance between the original location and each successive location (Papworth et al., 2012). A graph of NSD versus time gives a curve starting at the point of origin of a movement trajectory gradually reaching maximum NSD. NSD can remain constant or begin to drop as the animal returns to the point of origin where NSD = 0. Based on NSD, we calculated the time required for an animal to reach maximum displacement and return to the point of origin within the home range. The point of origin was selected randomly within the home range (approximately in the center of the home range) of the individual at a random time, calculated the revisit time, and considered it as one cycle. The time required to complete one such cycle was calculated. All movement parameters and analyses were carried out using *adehabitatLT* (Calenge, 2011) and animal movement tool (Signer et al., 2019) in program R 3.6.3 (R Core Team, 2020).

### 2.5 Understanding effect of anthropogenic factors on movement

Anthropogenic factors such as human population density, landuse, and road network have an adverse effect on animal movement through fragmented and disturbed habitats (Tucker et al., 2018). We estimated the human population density, landuse proportion, and road network within the home range of large carnivores. Home range was estimated using the Brownian Bridge Movement Model, BBMM (Bullard, 1999). BBMM is a widely used method that estimates the path of an animal's movement probabilistically from data recorded at brief intervals. BBMM quantifies the utilization distribution of an animal‐based on movement paths, accounts for temporal autocorrelation, and high data volumes (Fischer et al., 2013). The model approximates the movement path between two subsequent locations by applying a conditional random walk. We calculated utilization distribution for each individual at 50% and 95% isopleths to define the core area and home range, respectively, using the ArcMET extension tool (Wall, 2014) in ArcGIS 10.2 (Fischer et al., 2013; Laver & Kelly, 2008).

We used the human population density map (1 km resolution) available on the open‐source website (Stevens et al., 2015; http://www.worldpop.org.uk/). The landuse data of 1:25,000 scale was acquired from Bhuvan's open‐source website (NRSA, 2016; http://bhuvan.nrsc.gov.in/). The landuse maps were generated using “Resourcesat AWiFS” satellite imagery and classified Maharashtra into 13 landuse classes. We reclassified the original 13 classes into five major classes for analysis (Table 2). The road network data was obtained using Open Street Maps (Openstreetmap, 2015). We used primary and secondary roads for our assessment because of their significant impact on the movement of animals owing to higher traffic volumes (Saxena et al., 2020).

**TABLE 2:** Bhuvan's NRSA LULC original landuse classes and reclassified classes used for evaluation of the proportion of landuse within the home range

S. No.	Original class	Reclassified class
1	Builtup	Builtup
2	Kharif Crop	Agriculture
3	Rabi Crop	
4	Zaid Crop	
5	Double/Triple Crop	
6	Current Fallow	
7	Plantation	Forest
8	Evergreen Forest	
9	Deciduous Forest	
10	Degraded/Scrub Forest	
11	Wasteland	Grassland/Wasteland
12	Waterbody Max	Waterbody
13	Waterbody Min	

The effect of landuse, human population density, and road network on the hourly displacement was quantified in a regression framework. Each individual across species was considered as a single data point in the analysis. We used the percentage of each landuse class, average human population density, and road length in each animals’ home range as a predictor variable. We also compared the landuse and anthropogenic variables within the home range for the same species in different landscapes (tiger inside and outside PAs), social canids between wolf and dhole using *t* test. All the statistical analyses were carried out in R 3.6.3 (R Core Team, 2020).

### 2.6 Core area of large carnivores in heterogeneous landscape

Within home ranges, core areas are defined as exclusive areas of intensive use and likely contain features such as preferred foraging areas, dens, and resting sites (Ewer, 1968), facilitating many species to coexist. We computed the number, size, and perimeter of core areas across 4 large carnivore species. All home range metrics were calculated using the ArcMet tool (ArcGIS). For tigers, we compared the size and number of core areas of individuals of different sexes in varying levels of human disturbance. We also compared the core areas of wolf and dhole–two social canids of comparable body size but contrasting habitats. The significance of the results across species and habitats was tested using paired *t* test along with effect size (Zar, 1984).

## 3 RESULTS

A total of 48,646 fixes across 26 individuals of 4 large carnivore species were analyzed (Table 1). We examined the fundamental movement parameters, the impact of human footprint, and configuration of core areas of tiger, leopard, wolf, and dhole across a gradient of human disturbance.

### 3.1 Movement parameters of large carnivores

Inside PA, the average hourly displacement of tiger and leopard was 196.23 ± 49.93 m/hr and 99.34 ± 27.9 m/hr, respectively, whereas dhole moved an average of 259.92 ± 49.68 m/hr. Outside PA, the mean tiger displacement was 312.20 ± 136.76, and wolf moved an average of 637.70 ± 87.80 m/hr (Table 3).

**TABLE 3:** Displacement of 4 large carnivores across different habitat types in India

Species	Habitat/system	Behavioral trait	Mean Displacement (m/hr)	Mean displacement during day (m/hr)	Mean displacement during night (m/hr)
Tiger PA	Dry Deciduous Forest (PA)	Solitary	196.23 ± 49.93	174.62 ± 48.6	218 ± 53.58
Tiger Outside PA	Dry Deciduous Forest and Agriculture Interface	Solitary	312.20 ± 136.76	241.11 ± 75.33	377.30 ± 196.85
Leopard	Dry Deciduous Forest (PA)	Solitary	99.34 ± 27.9	101.51 ± 52.48	91.34 ± 11.68
Dhole	Dry Deciduous Forest (PA)	Social	259.92 ± 49.68	337.92 ± 133.97	191.62 ± 66.97
Wolf	Human‐Dominated Grassland‐Agriculture Mosaic	Social	637.70 ± 87.8	471.09 ± 165.62	819.33 ± 154.22

Mean hourly displacement of tigers was found to be higher outside PA (312.20 ± 136.76 m/hr) than PA (196.23 ± 49.93 m/hr) however, they were significantly not different (*p* = .06; Glass's ∆ = 2.37). For tigers inside and outside PAs, mean hourly displacement varied significantly between day (174.62 ± 48.6 m/hr; *p* = .0007; Glass's ∆ = 0.89) and night (218.0 ± 53.58 m/hr; *p* = .03; Glass's ∆ = 1.8) with higher displacement during night across the landscape. Among sexes, mean displacement per hour of tigers varied with males having larger displacement (252.54 ± 117.59 m/hr) than females (200.42 ± 43.87 m/hr). Moreover, both the sexes showed longer displacement during night than day. Leopards showed the least variation in mean displacement through day and night (101.51 ± 52.48 m/hr and 91.34 ± 11.68 m/hr) respectively. The dhole which inhabits forested areas showed higher displacement during daytime (337.92 ± 133.97 m/hr) as compared to night (191.62 ± 66.97 m/hr), and the difference was found significant (*p* = .03; Glass's ∆=1.09). The wolves showed higher mean displacement during night (819.33 ± 154.22 m/hr) as compared to day (471.09 ± 165.62 m/hr), and significant difference was found (*p* = .05; Glass's ∆=2.1).

Based on NSD, all species across the landscape exhibited a confined movement pattern. The tiger outside PA took 141.4 ± 44.77 hr to complete one cycle (point of origin—maximum displacement—point of origin), whereas tiger inside PA (208.4 ± 167.7 hr) took 32.14% higher time than outside PA. For leopards, the time to complete each cycle was found to be maximum (1,258.50 ± 485.59 hr). Dholes and wolves took similar time to complete one cycle to cover their home ranges (204.915 ± 83.71 hr and 229.76 ± 111.6 hr respectively) (Table 4).

**TABLE 4:** Based on NSD, time required for species to complete one cycle from point of origin to maximum displacement and back as a proxy for time taken to cover one home range circuit

Species	Number of individuals (*n*)	Number of cycles	Range to complete one cycle (h)	Time to complete one cycle (h)
Tiger (PA)	9	99	15–1,159	208.4 ± 167.7
Tiger (Outside PA)	5	42	21–620	141.4 ± 44.77
Leopard	3	8	216–3,168	1,258.50 ± 485.59
Dhole	5	28	27–708	204.915 ± 83.71
Wolf	4	17	60–480	229.76 ± 111.6

### 3.2 Effect of anthropogenic factors on movement of large carnivores

The hourly displacement of the large carnivores varied from 99.35m/h for leopards to 637.7m/h. When we modeled the hourly displacement with the landuse classes, human density, and road length in the home range of the individual, most of the variance is explained by two landuse classes (*F*_2,22_ = 4.582, *p* = .021; agriculture, *r* = .52, *p* = .009 and wasteland/grassland, *r* = .49, *p* = .013). Both variables showed positive association with hourly displacement (Table 5). For tigers, forest area within the home range was not significantly different between PAs and outside PAs (*p* forest = .06) whereas, agriculture and road length were found to be significantly different (*p* agriculture = .03, *p* roads = .02). For the social canids, wolf, and dhole all the habitat variables in the home range were found to be significantly different (*p* human density < .001, *p* roads = .005, *p* agriculture = .04, *p* forest < .001, *p* wasteland/grassland = .008, *p* waterbody = .005).

**TABLE 5:** Description of model parameters used to evaluate the effect of overall hourly displacement of all individuals and regression results of individual models

Model parameters	Degrees of freedom	AIC	Adjusted *R* ^2^
Human population density in HR + agriculture area in HR + wasteland/grassland in HR	4	58.982	0.401
Human population density in HR + agriculture area in HR + wasteland/grassland in HR + road length in HR	5	59.703	0.407
Human population density in HR + agriculture area in HR + wasteland/grassland in HR + road length in HR + waterbody in HR	6	61.603	0.381
Human population density in HR + agriculture area in HR + wasteland/grassland in HR + road length in HR + waterbody in HR + Forest area in HR	7	63.551	0.348
Human population density in HR + agriculture area in HR + wasteland/grassland in HR + builtup area in HR + Forest area in HR + waterbody in HR + road length in HR	9	321.071	0.376

### 3.3 Core area of large carnivores in heterogeneous landscape

**FIGURE 2:**
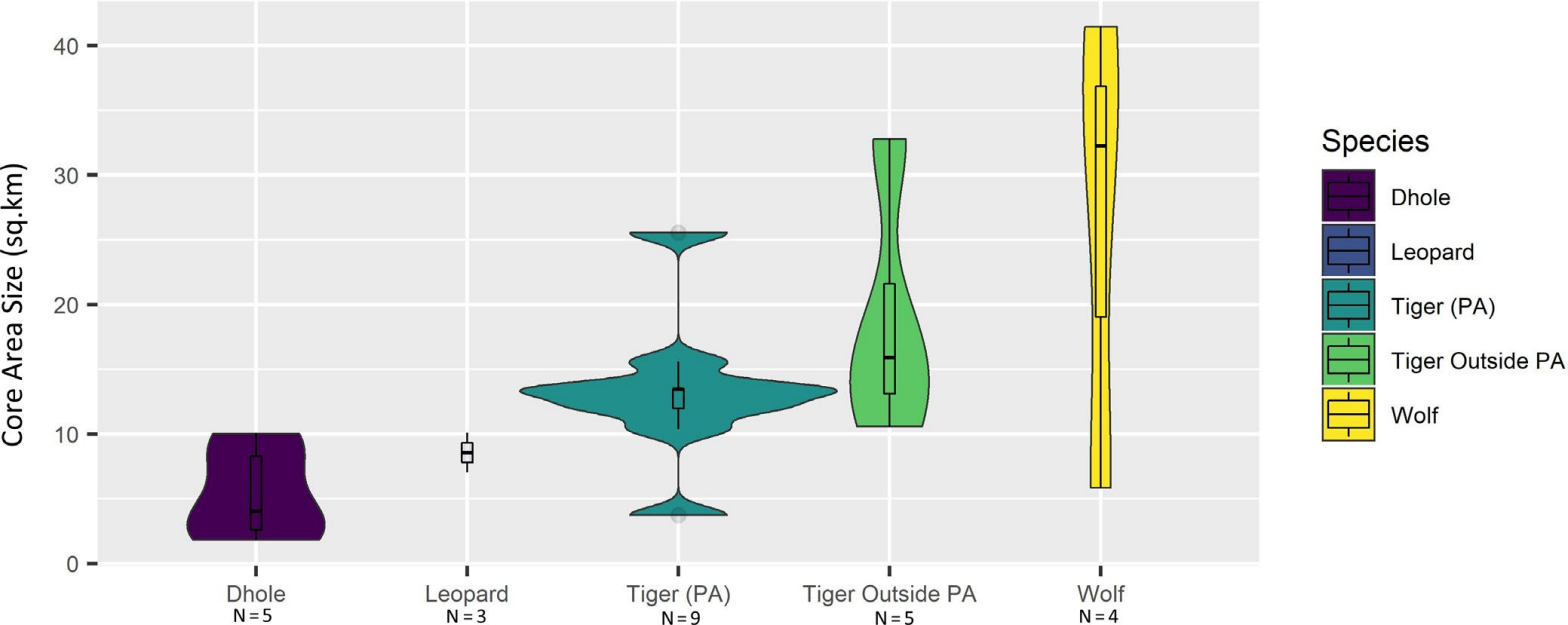
Violin plot indicating the distribution of the range of the core area size, median (black center line), and spread of the data (black rectangle) for four large carnivores in India

All carnivores showed multiple areas of intensive use or cores in their home ranges. The mean number of core areas per individual was not significantly different among species (Table 6). The range of the core area sizes was greater for species outside PAs (tiger: 0.68–29.31 km^2^ and wolf: 0.55–25.84 km^2^) in human‐altered landscapes. For dhole (1.37–7.04 km^2^) and leopard (0.65–15.67 km^2^), the spread was found lowest inside PA (Figure 2).

**TABLE 6:** Mean number, size, and perimeter of core areas of four large carnivores and tigers across sex and between inside and outside protected area in India

Species	Mean no. of core areas	Mean core area size (km^2^)	Mean core area perimeter (km)	Total perimeter (km)
Tiger PA	2 ± 1.80	5.99 ± 5.50	14.97 ± 10.56	29.9
Tiger outside PA	3.25 ± 1.70	5.6 ± 7.77	12.53 ± 10.04	40.7
Dhole	2.2 ± 1.7	2.21 ± 1.6	8.17 ± 4.48	18.0
Wolf	2.33 ± 1.52	11.37 ± 9.96	15.08 ± 8.33	35.1
Leopard	2 ± 1.41	3.85 ± 2.74	11.92 ± 7.23	23.8
Tiger outside PA (Male)	3.33 ± 2.08	5.94 ± 8.72	13.02 ± 11.03	43.4
Tiger PA (Male)	3.25 ± 2.21	4.62 ± 5.14	12.05 ± 10.53	39.2
Tiger 0utside PA (Female)	3	4.46 ± 4.16	10.93 ± 7.25	32.8
Tiger PA (Female)	1 ± 0	11.23 ± 5.79	19.63 ± 6.57	19.6

The number of core areas of tigers inside and outside PAs was significantly different (*p* = .03; Glass's ∆ = 1.41), whereas the difference in size of core areas was not significant (*p* = .43; Glass's ∆ = 0.07). Although the median value of core area size was higher for tigers inside PAs (4.0 km^2^) in comparison to the tigers outside PAs (1.53 km^2^), the range of core area size was greater for tigers outside PAs (0.55–25.84 km^2^) than inside (0.65–15.67km^2^) (Table 6). The two social canids, dhole and wolf, have a comparable body size, but the size of core areas was completely different. The number of core areas for both canids did not differ significantly (*p* = .46; Glass's ∆ = 0.07), but core area sizes were significantly different (*p* = .004; Glass's ∆ = 5.7). Core areas of dholes were smaller with narrow ranges (0.6–5.05 km^2^), whereas wolves exhibited a wide range of core sizes (0.68–29.31 km^2^) similar to tigers outside PAs.

## 4 DISCUSSION

### 4.1 Movement of large carnivores across human‐dominated landscapes

Large carnivore species living outside PAs exhibited greater mean displacement (25.29%) than the species inside PAs with a single exception of the dhole. Dholes moved with higher speeds (i.e., with longer step lengths) among the 3 large carnivores sharing a similar habitat inside PAs. Predominantly occurring in a human‐dominated landscape, wolves showed the highest movement among all 4 carnivores, whereas the leopards in natural areas showed the least. We also found tigers outside PAs moved at higher speeds than inside PAs. Our result on wolves and tigers outside PAs ties well with previous studies on similar species in human‐dominated landscapes like cougars (*Puma concolor*) and lions (*Panthera leo persica*) that exhibited higher speeds while traversing through fragmented areas to reduce time spent in multiple‐use areas (Kertson et al., 2011; Valeix et al., 2012).

Across sexes, both male and female tigers traveled more during night than day. Male tigers traveled faster than female tigers owing to larger home ranges and longer distance to cover in habitat matrix. As males exhibit multiple core areas in human‐altered landscapes, the movement rate to travel between core areas was high. The leopard movement was found lowest 99.34 ± 27.9 among all carnivores within the PAs with less difference between day and night movements. This may be because leopards survive in the presence of large predators like tigers and pack‐living dholes that make up for their size in numbers. Intense intraguild competition has driven leopards to the boundaries of protected areas where they are faced with increased human pressures (Azlan & Sharma, 2006; Carter et al., 2015; Odden et al., 2010; Seidensticker et al., 1990). Moreover, leopards also took the highest time (1,258.50 ± 485.59 hr) to return from the point of maximum displacement to the point of origin within the home range. Under such circumstances, leopards travel from one core area to another and spend more time in such core areas. This strategy may enable them to coexist with large carnivores and humans. Interestingly, tigers outside PAs took comparatively lesser time (141.4 ± 44.77 hr) to cover their home range than tigers inside PAs (208.4 ± 167.7 hr) even though their home ranges were twice the size. This may be because the tigers in human‐disturbed areas move faster owing to the presence of habitat matrix between core areas, which enables them to cover larger areas in a shorter time.

### 4.2 Effects of human footprint on movement of large carnivores

**FIGURE 3:**
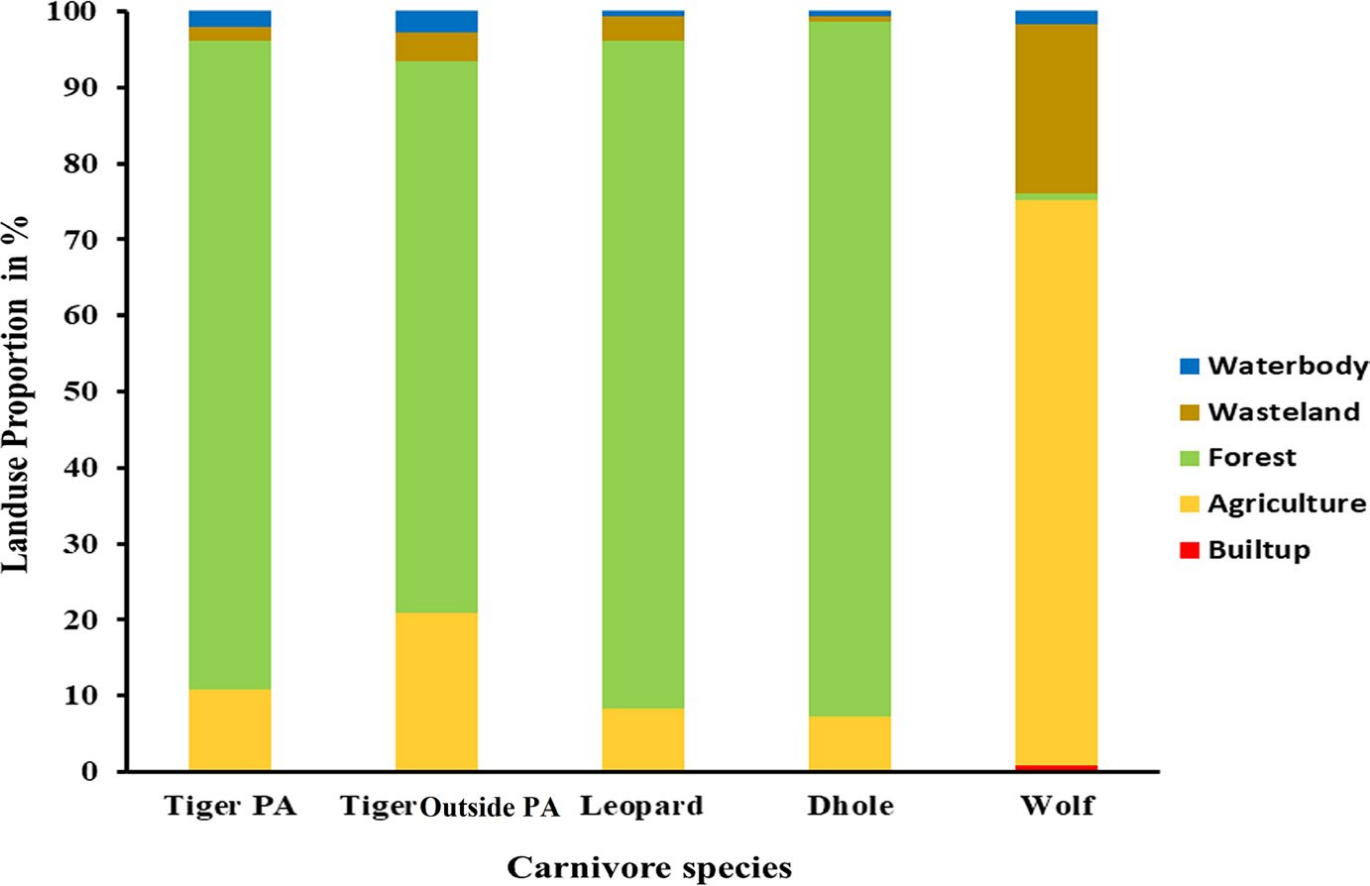
Landuse proportion within the home range of four large carnivores in India. Data from Bhuvan's LULC (http://bhuvan.nrsc.gov.in/) was used to classify home ranges

As human activities increase, the collateral loss of habitat and biodiversity is accompanied by a change in the movement of animals through fragmented landscapes (Tucker et al., 2018). Landscape structure affects movement parameters because different cover types in the landscape offer different levels of risk and benefit. Landuse types across home ranges of large carnivore species were not significantly different with the single exception of wolves, which live primarily in grasslands and human‐altered landscapes (Figure 3). Historically, wolves adapted to live in human‐dominated landscapes as they evolved near humans (Anderson, 2018). Moreover, our results indicate that the wolves move faster in human‐dominated landscapes may be to negotiate human pressures and avoid them as much as possible within their large home ranges.

The displacement of tigers outside PA was 62.85% higher than inside PA, though it did not differ significantly. Parameters supposed to influence the hourly displacement such as population density, landuse proportion, and road length was significantly different (*p* = .01) within and outside protected area. The forest outside PAs is fragmented with high human density and road network, which may explain the larger home ranges of tigers outside PAs (Habib, Nigam, et al., 2018). To negotiate this fragmented landscape, tigers outside PAs also move at higher speeds than inside PAs.

We also compared the movement parameters of two social carnivores; wolf present in a human‐dominated landscape and dhole inhibiting protected area and found that the hourly displacement of wolf was 62.90% higher than dholes. The parameters influencing the hourly displacement such as population density, landuse proportion, and road length were also significantly different (*p* = .04) between the home ranges. Ecologically, as the percent of agriculture in an individual's home range increases, the individual has to move more to exploit resources in the human‐dominated landscape. Moreover, area of grassland is positively related, as large carnivores like wolf are known to prefer grassland habitat and showed highest hourly displacement.

**FIGURE 4:**
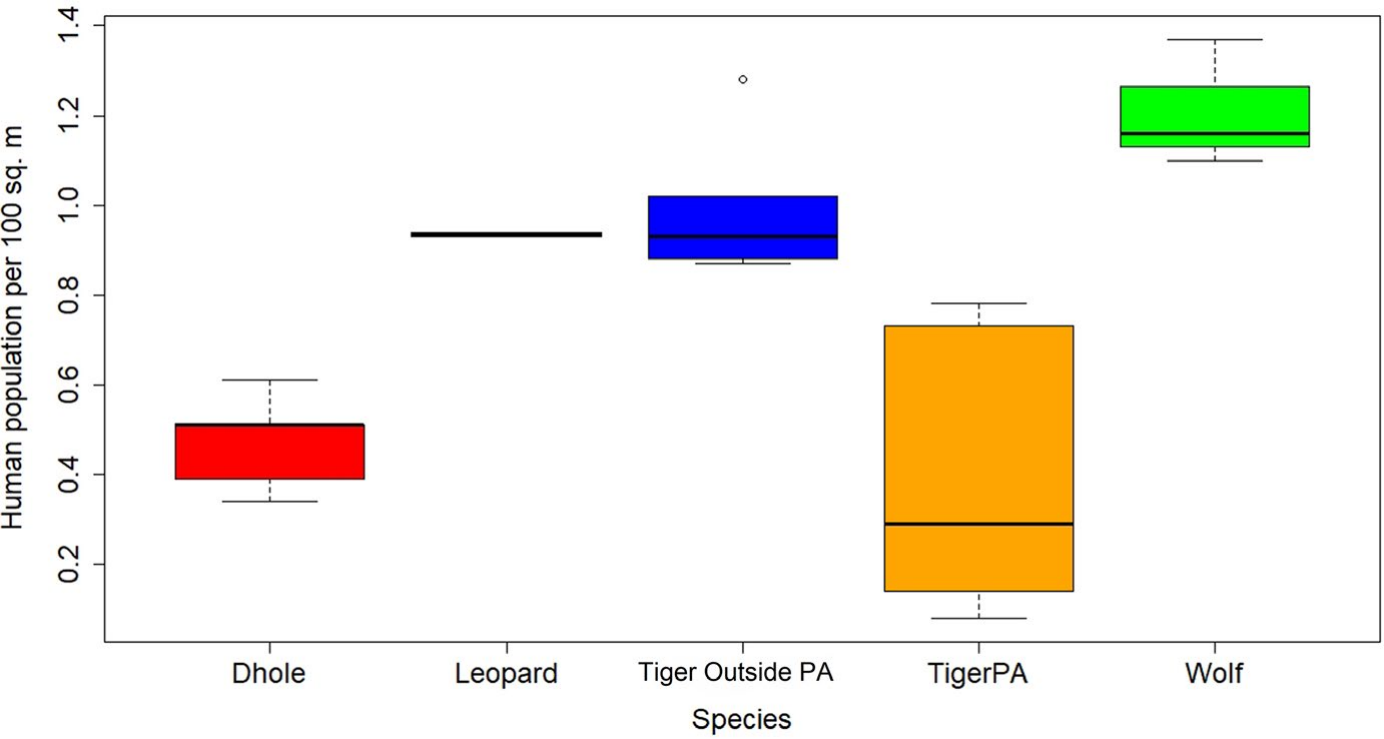
Human population density within the home range of four large carnivores in India

**FIGURE 5:**
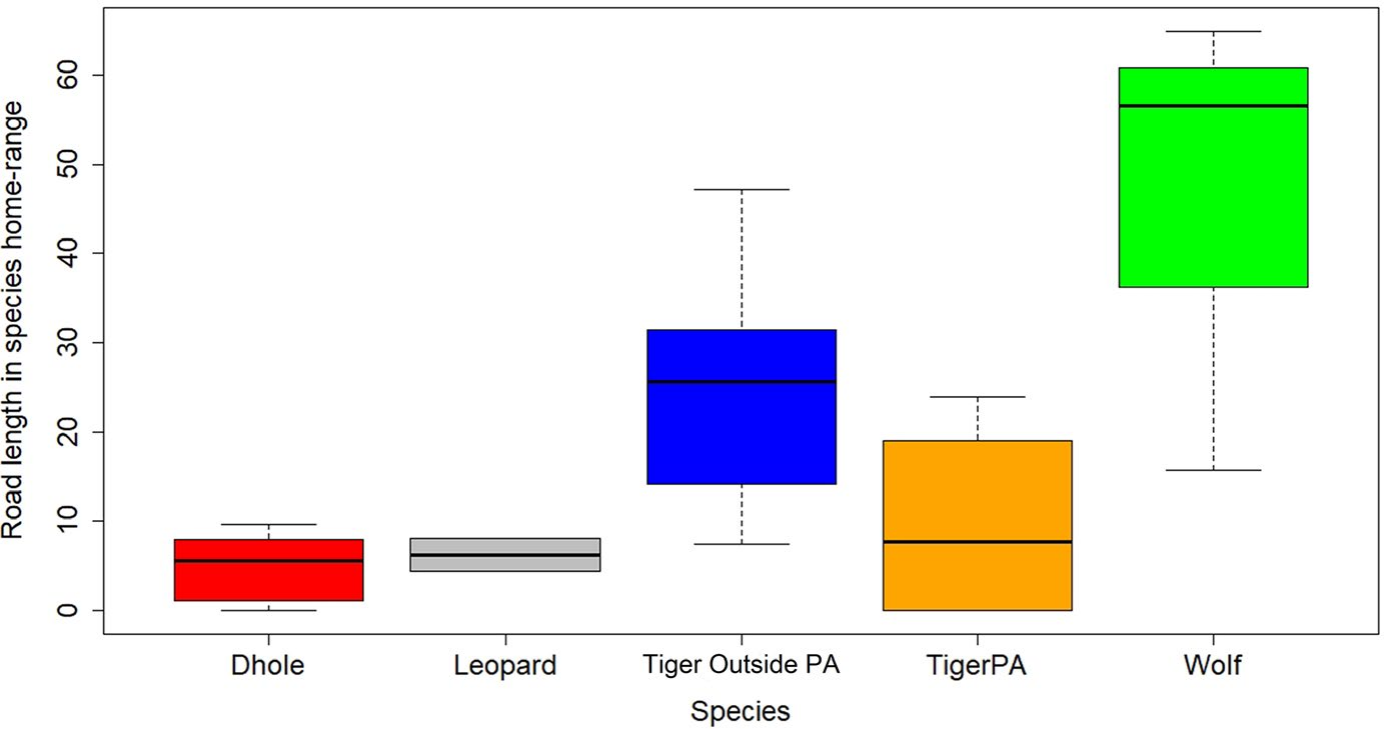
Road length (km) within the home range of four large carnivores in India

We examined the proportion of human population and road length inside the home ranges of the 4 large carnivores in our study areas. As expected, home range of wolves consisted of relatively high human density followed by leopard and tigers outside PAs. Within PAs, dholes showed higher human population pressure (0.51 human/100 km^2^) than tigers (0.29 human/100 km^2^) in their home ranges (Figure 4). This may be because dholes establish intensive use areas near PA fringes as a strategy to avoid large predators and competition (Ghaskadbi & Habib, 2019). Across our study sites, the home range of wolves had the maximum network of roads (56.6 km), followed by tigers outside PAs (25.7 km). The home range of dholes showed the least length of roads (5.5 km) (Figure 5). All carnivores had primary roads passing through their home ranges, but the disturbance caused by them need not be the same. This is because the roads inside PAs are nonfunctional and only used for limited tourist activity and management.

We also compared the landuse class within tiger home ranges, which suggested that the proportion of forest cover was not significantly different, whereas agriculture outside and inside PAs differed significantly. It is worth discussing that home ranges of tigers outside PAs were primarily forest areas (72.72%). Tigers outside PA uses fragmented landscape and form multiple core areas primarily dominated by forest areas to avoid humans and meet their basic ecological requirements.

### 4.3 Core area of large carnivores in heterogeneous landscape

Core areas of animals have been studied to address a wide range of research queries (Hooten et al., 2008) such as social information transmission (Darden et al., 2008), interspecific competition (Neale & Sacks, 2001), trophic cascades (Prange & Gehrt, 2007), habitat selection (Chamberlain et al., 2003), reproductive success (Thompson et al., 2007) and territorial defense (Darden & Dabelsteen, 2008). Our study reports multiple areas of intensive use or cores for all the 4 carnivores across the landscape (Table 6). The number and size of core areas across species did not show a significant difference, but the ranges were different. For species surviving in human‐altered landscapes like the wolf and tigers outside PAs, the range of core area size was the greatest, whereas it was the least for the dholes.

For tigers outside PA, we found core areas with a larger perimeter than tiger inside PAs. This may be because of the high level of fragmentation and human pressure. The core area with larger perimeter for tiger and wolf outside PA indicates higher chances of exposure to human‐induced effect leading to an increase in human–animal interaction. The female tiger in PA had only one core area, whereas female tigers outside PA had multiple core areas with larger perimeter (Table 6). This result explains the possibility that female tigers outside PAs are more prone to conflict due to their higher energy demand and greater perimeter owing to more chance of interaction with humans.

## 5 CONSERVATION IMPLICATIONS

Across the globe, large carnivore conservation is a challenge owing to the habitat loss and fragmentation of natural areas with rapidly growing human populations. In India, the conservation of large carnivores is interlaced with various political, socioeconomic, and emotional issues, which further complicates this challenge. Increasingly, wildlife is compelled to coexist with humans in highly modified landscapes, highlighting the need for planned and coordinated interdisciplinary efforts. Integrating movement ecology in landscape management and policymaking is a desirable approach as it provides insights into how animals are affected by human footprint and the implications on their ecology and conservation. With great advances being made across the world in the field of movement ecology, India is only beginning to take the initial steps into the field.

The novel findings of the large‐scale study on the movement ecology of 4 large carnivores of India will have major implications on their conservation and management in the country. They may even guide developing countries with high human and carnivore densities in conservation planning and management and serve as cautionary learning for countries where the densities of populations may increase in the future. If large carnivores are to coexist with humans, there needs to be an understanding of how animals move inside PAs and the adaptations they exhibit outside PAs to survive in the matrix in between. The use and extent of corridors need to be informed by real‐time knowledge of animal motion and navigation capacities if we are to safeguard the sensitive connections between the PAs. The authors are aware of the limitations of this study compared to long‐term and large‐scale radio‐collaring studies across the globe. However, our study can be a suitable starting point for further comparative studies to understand the extent to which large carnivores can negotiate landscapes and adapt to survive.

## CONFLICT OF INTEREST

The authors declare that they have no competing interests.

## AUTHOR CONTRIBUTION

**Bilal Habib:** Conceptualization (lead); Funding acquisition (lead); Investigation (lead); Methodology (lead); Project administration (lead); Resources (lead); Software (lead); Supervision (lead); Validation (lead); Visualization (lead); Writing‐review & editing (lead). **Pallavi Ghaskadbi:** Data curation (equal); Formal analysis (equal); Methodology (equal); Writing‐original draft (equal); Writing‐review & editing (equal). **Shaheer Khan:** Data curation (equal); Formal analysis (equal); Methodology (equal); Writing‐original draft (equal); Writing‐review & editing (equal). **Zehidul Hussain:** Data curation (equal); Formal analysis (equal); Methodology (equal); Writing‐original draft (equal); Writing‐review & editing (equal). **Parag Nigam:** Data curation (equal); Investigation (equal); Methodology (equal); Resources (equal); Validation (equal); Writing‐review & editing (equal).

## ETHICAL APPROVAL

All the four species were captured following standard and approved protocols after due permission from the Ministry of Environment, Forests and Climate Change, Government of India, and Maharashtra Forest Department. The species‐specific permit details are as follows: Tigers and Leopards: MOEF&CC – F. No. 1‐36/2014‐WL‐I/05.09.2014; F. No. 1‐22/2015/WL/09.10.2015; MFD – SPP‐144/13.10.2014; SPP‐04/01.01.2016, Wolf: MOEF&CC – F. No. 1‐69/2017‐WL/16.05.2017; MFD – SPP‐15/01.06.2017, Dhole ‐ MFD – SPP‐12/05.11.2016.

## Data Availability

Because of conservation reasons, the authors are unable to share the location data for the carnivore species.

## References

[ece37156-bib-0001] 19th Livestock Census (2012). All India Report. Ministry of Agricultural Department of Animal Husbandry, Dairying and Fisheries, Krishi Bhawan, New Delhi.

[ece37156-bib-0002] Abrahms, B. , Seidel, D. P. , Dougherty, E. , Hazen, E. L. , Bograd, S. J. , Wilson, A. M. , McNutt, J. W. , Costa, D. P. , Blake, S. , Brashares, J. S. , & Getz, W. M. (2017). Suite of simple metrics reveals common movement syndromes across vertebrate taxa. Movement Ecology, 5(1), 12 10.1186/s40462-017-0104-2 28580149 PMC5452391

[ece37156-bib-0003] Acharya, B. B. (2007). The ecology of the dhole or Asiatic wild dog (*Cuon alpinus*) in Pench Tiger Reserve, Madhya Pradesh. PhD Thesis. Saurashtra University: Rajkot, India.

[ece37156-bib-0004] Andersen, G. E. , Johnson, C. N. , Barmuta, L. A. , & Jones, M. E. (2017). Use of anthropogenic linear features by two medium‐sized carnivores in reserved and agricultural landscapes. Scientific Reports, 7(1), 1–11. 10.1038/s41598-017-11454-z 28912508 PMC5599595

[ece37156-bib-0005] Anderson, E. N. (2018). The First Domestication: How wolves and humans coevolved. By Raymond Pierotti and Brandy R. Fogg. 2017. Yale University Press, New Haven. 326 pp. Ethnobiology Letters, 9(2); 247–249.

[ece37156-bib-0006] Azlan, J. M. , & Sharma, D. S. K. (2006). The diversity and activity patterns of wild felids in a secondary forest in Peninsular Malaysia. Oryx, 40, 36–41. 10.1017/S0030605306000147

[ece37156-bib-0007] Basic Road Statistics of India (2016). Ministry of road transport and highways, transport research wing, New Delhi (pp. 77). http://www.indiaenvironmentportal.org.in/

[ece37156-bib-0008] Bullard, F. (1999). Estimating the home range of an animal: a Brownian bridge approach. MSc thesis. Johns Hopkins University.

[ece37156-bib-0009] Cagnacci, F. , Boitani, L. , Powell, R. A. , & Boyce, M. S. (2010). Animal ecology meets GPS‐based radiotelemetry: A perfect storm of opportunities and challenges. Philosophical Transactions of the Royal Society B., 365(1550), 2157–2162. 10.1098/rstb.2010.0107 PMC2894970 20566493

[ece37156-bib-0010] Calenge, C. (2011). Analysis of animal movements in R: The adehabitatLT package. R Foundation for Statistical Computing.

[ece37156-bib-0011] Carter, N. , Jasny, M. , Gurung, B. , & Liu, J. (2015). Impacts of people and tigers on leopard spatiotemporal activity patterns in a global biodiversity hotspot. Global Ecology and Conservation, 3, 149–162. 10.1016/j.gecco.2014.11.013

[ece37156-bib-0012] Chamberlain, M. J. , Leopold, B. D. , & Conner, L. M. (2003). Space use, movements and habitat selection of adult bobcats (*Lynx rufus*) in central Mississippi. The American Midland Naturalist., 149(2), 395–405.

[ece37156-bib-0013] Chundawat, R. S. , Gogate, N. S. , & Johnsingh, A. J. T. (1999). Tigers in Panna: Preliminary results from an Indian tropical dry forest In J. Seidensticker , S. Christie , & P. Jackson (Eds.), Riding the Tiger: Tiger conservation in human‐dominated landscape. Cambridge University Press.

[ece37156-bib-0014] Chundawat, R. S. , Sharma, K. , Gogate, N. , Malik, P. K. , & Vanak, A. T. (2016). Size matters: Scale mismatch between space use patterns of tigers and protected area size in a Tropical Dry Forest. Biological Conservation, 197, 146–153. 10.1016/j.biocon.2016.03.004

[ece37156-bib-0015] Clobert, J. , Le Galliard, J. F. , Cote, J. , Meylan, S. , & Massot, M. (2009). Informed dispersal, heterogeneity in animal dispersal syndromes and the dynamics of spatially structured populations. Ecology Letters, 12(3), 197–209. 10.1111/j.1461-0248.2008.01267.x 19170731

[ece37156-bib-0016] Darden, S. K. , & Dabelsteen, T. (2008). Acoustic territorial signalling in a small, socially monogamous canid. Animal Behavior., 75(3), 905–912. 10.1016/j.anbehav.2007.07.010

[ece37156-bib-0017] Darden, S. K. , Steffensen, L. K. , & Dabelsteen, T. (2008). Information transfer among widely spaced individuals: Latrines as a basis for communication networks in the swift fox? Animal Behavior, 75(2), 425–432. 10.1016/j.anbehav.2007.05.007

[ece37156-bib-0018] Evans, M. J. , Hawley, J. E. , Rego, P. W. , & Rittenhouse, T. A. (2019). Hourly movement decisions indicate how a large carnivore inhabits developed landscapes. Oecologia, 190(1), 11–23. 10.1007/s00442-018-4307-z 30506304

[ece37156-bib-0019] Ewer, R. F. (1968). Ethology of mammals. Legos Press.

[ece37156-bib-0020] Fahrig, L. (2007). Non‐optimal animal movement in human‐altered landscapes. Functional Ecology., 21(6), 1003–1015. 10.1111/j.1365-2435.2007.01326.x

[ece37156-bib-0021] Fischer, J. W. , Walter, W. D. , & Avery, M. L. (2013). Brownian Bridge Movement Models to characterize birds' home ranges. Condor, 115(2), 298–305. 10.1525/cond.2013.110168

[ece37156-bib-0022] Ghaskadbi, P. , & Habib, B. (2019). A dhole lot of movement: Spatial ecology of the endangered Asiatic wild dog, dhole in Tadoba Andhari Tiger Reserve, India. Paper presented at the British Ecological Society Annual Meeting, Belfast.

[ece37156-bib-0023] Ghaskadbi, P. , Habib, B. , & Qureshi, Q. (2016). A whistle in the woods: An ethogram and activity budget for the dhole in central India. Journal of Mammalogy, 97(6), 1745–1752. 10.1093/jmammal/gyw141

[ece37156-bib-0024] Goodrich, J. M. , Miquelle, D. G. , Smirnov, E. N. , Kerley, L. L. , Quigley, H. B. , & Hornocker, M. G. (2010). Spatial structure of Amur (Siberian) tigers (*Panthera tigris altaica*) on Sikhote‐Alin biosphere Zapovednik, Russia. Journal of Mammalogy, 91, 737–748. 10.1644/09-MAMM-A-293.1

[ece37156-bib-0025] Habib, B. (2007). Ecology of Indian wolf (Canis lupus pallipes Sykes 1831) and modeling its potential in the Great Indian Bustard Sanctuary, Maharashtra, India (p. 274). PhD thesis, Department of Wildlife Sciences Aligarh Muslim University.

[ece37156-bib-0026] Habib, B. , Ghaskadbi, P. , Nigam, P. , Modi, S. , Kumar, P. S. , Sharma, K. , Singh, V. , Kumar, B. , Tripathi, A. , Kothandaraman, H. , Yellaboina, S. , Baghel, D. S. , & Mondol, S. (2018). The Cuon Enigma: Genome survey and comparative genomics of the endangered Dhole (Cuon alpinus). BioRxiv, 443119, 10.1101/44311

[ece37156-bib-0027] Habib, B. , & Kumar, S. (2007). Den shifting by wolves in semi‐wild landscapes in the Deccan Plateau, Maharashtra, India. Journal of Zoology., 272(3), 259–265. 10.1111/j.1469-7998.2006.00265.x

[ece37156-bib-0028] Habib, B. , Nigam, P. , Mondal, I. , Ghaskadbi, P. , & Hussain, Z. (2017). Ensuring safety in the killer fields: Identifying potential villages for measures to reduce electrocution of Tigers and associated species in Eastern Vidarbha Landscape, Maharashtra, India. Wildlife Institute of India, Dehradun, National Tiger Conservation Authority and Maharashtra Forest Department Report; pp 115.

[ece37156-bib-0029] Habib, B. , Nigam, P. , Mondol, I. , Ghaskadbi, P. , & Hussain, Z. (2018). Forest fragments in Eastern Vidarbha Landscape, Maharashtra. The Tig – Saw Puzzle (pp. 37). Wildlife Institute of India and Maharashtra Forest Department, TR No. 2018/17.

[ece37156-bib-0030] Haddad, N. M. (1999). Corridor use predicted from behaviors at habitat boundaries. The American Naturalist, 153(2), 215–227. 10.1086/303163 29578761

[ece37156-bib-0031] Hayward, M. W. , Lyngdoh, S. , & Habib, B. (2014). Diet and prey preferences of dholes (*Cuon alpinus*): Dietary competition within Asia's apex predator guild. Journal of Zoology, 294(4), 255–266.

[ece37156-bib-0032] Hayward, M. W. , & Slotow, R. (2009). Temporal partitioning of activity in large African carnivores: Tests of multiple hypotheses. South African Journal of Wildlife Research, 39(2), 109–125. 10.3957/056.039.0207

[ece37156-bib-0033] Hooten, M. B. , Wilson, R. R. , & Shivik, J. A. (2008). Hard Core or Soft Core: On the characterization of animal space use. In Joint Statistical Meeting, Denver, Colorado.

[ece37156-bib-0034] Jethva, B. D. (2003). Feeding ecology and habitat needs of wolves (Canis lupus pallipes) in the Bhal area of Gujarat (pp. 95). PhD Dissertation, Forest Research Institute Deemed University, Dehradun, India.

[ece37156-bib-0035] Jhala, Y. V. (1991). The habitat and population dynamics of wolves and blackbuck in Velavadar National Park, Gujarat, India (p. 236). PhD thesis. Virginia Polytechnic Institute and State University, Blacksburg, Virginia.

[ece37156-bib-0036] Jhala, Y. V. (2000). Human‐conflict in India. Abstract in “Beyond 2000: realities of global wolf restoration”. Symposium, Duluth, MN, USA, February 23–26.

[ece37156-bib-0037] Jhala, Y. V. , Quereshi, Q. , Vettakevan, J. , Borah, J. , & Kumar, U. (2010). Intensive Population Monitoring and Study of Tiger Dispersal in Kanha Tiger Reserve (Phase IV). Final Report. Wildlife Institute of India, Dehradun, India.

[ece37156-bib-0038] Jhala, Y. V. , Qureshi, Q. , & Nayak, A. K. (Eds.) (2020). Status of tigers, co‐predators and prey in India, 2018. National Tiger Conservation Authority, Government of India, New Delhi, and Wildlife Institute of India, Dehradun. 81–85496.

[ece37156-bib-0039] Johnsingh, A. J. T. (1980). Ecology and Behaviour of the Dhole or Indian Wild Dog, Cuon Alpinus Pallas 1811, with Special Reference to Predator: Prey Relations at Bandipur. WWF Report.

[ece37156-bib-0040] Johnsingh, A. J. T. (1986). Diversity and conservation of carnivorous mammals in India. Proceedings of Indian Academy of Sciences (Animal Sciences/Plant Sciences) Supplement, 73–89.

[ece37156-bib-0041] Karanth, K. U. , & Sunquist, M. E. (2000). Behavioural correlates of predation by tiger (*Panthera tigris*), leopard (*Panthera pardus*) and dhole (*Cuon alpinus*) in Nagarahole, India. Journal of Zoology., 250(2), 255–265. 10.1111/j.1469-7998.2000.tb01076.x

[ece37156-bib-0042] Kays, R. , Crofoot, M. C. , Jetz, W. , & Wikelski, M. (2015). Terrestrial animal tracking as an eye on life and planet. Science, 348(6240), 2478 10.1126/science.aaa2478 26068858

[ece37156-bib-0043] Kerley, L. L. , Goodrich, J. M. , Miquelle, D. G. , Smirnov, E. N. , Quigley, H. B. , & Hornocker, M. G. (2002). Effects of roads and human disturbance on Amur Tigers. Conservation Biology, 16, 97–108. 10.1046/j.1523-1739.2002.99290.x 35701953

[ece37156-bib-0044] Kertson, B. N. , Spencer, R. D. , Marzluff, J. M. , Hepinstall‐Cymerman, J. , & Grue, C. E. (2011). Cougar space use and movements in the wildland–urban landscape of western Washington. Ecological Application, 21(8), 2866–2881.

[ece37156-bib-0045] Kozakai, C. , Yamazaki, K. , Nemoto, Y. , Nakajima, A. , Umemura, Y. , Koike, S. , Goto, Y. , Kasai, S. , Abe, S. , Masaki, T. , & Kaji, K. (2013). Fluctuation of daily activity time budgets of Japanese black bears: Relationship to sex, reproductive status, and hard‐mast availability. Journal of Mammalogy, 94(2), 351–360. 10.1644/11-MAMM-A-246.1

[ece37156-bib-0046] Laver, P. N. , & Kelly, M. J. (2008). A critical review of home range studies. The Journal of Wildlife Management, 72(1), 290–298. 10.2193/2005-589

[ece37156-bib-0047] Leblond, M. , St‐Laurent, M. H. , & Côté, S. D. (2016). Caribou, water, and ice–fine‐scale movements of a migratory arctic ungulate in the context of climate change. Movement Ecology, 4(1), 14 10.1186/s40462-016-0079-4 27099756 PMC4837602

[ece37156-bib-0048] Linnell, J. D. , Swenson, J. E. , & Anderson, R. (2001). Predators and people: Conservation of large carnivores is possible at high human densities if management policy is favourable. Animal Conservation Forum, 4(4), 345–349.

[ece37156-bib-0049] Miquelle, D. G. , Nikolaev, I. , Goodrich, J. M. , Litvinov, B. , Smirnov, E. , & Suvorov, E. (2005). Searching for the coexistence recipe: A case study of conflicts between people and tigers in the Russian Far East In R. Woodroffe , S. Thirgood , & A. R. Rabinowitz (Eds.), People and wildlife: Conflict or coexistence (pp. 305–322). Cambridge University Press.

[ece37156-bib-0050] Modi, S. , Mondol, S. , Ghaskadbi, P. , Hussain, Z. , Nigam, P. , & Habib, B. (2018). Noninvasive DNA‐based species and sex identification of Asiatic wild dog (*Cuon alpinus*). Journal of Genetics, 97(5), 1457–1461. 30555094

[ece37156-bib-0051] Mondal, I. , Habib, B. , Talukdar, G. , & Nigam, P. (2016). Triage of means: Options for conserving tiger corridors beyond designated protected lands in India. Frontiers Ecology and Evolution, 4, 133.

[ece37156-bib-0052] Morales, J. M. , Moorcroft, P. R. , Matthiopoulos, J. , Frair, J. L. , Kie, J. G. , Powell, R. A. , Merrill, E. H. , & Haydon, D. T. (2010). Building the bridge between animal movement and population dynamics. Philosophical Transactions of the Royal Society of London. Series B, Biological Sciences, 365(1550), 2289–2301. 10.1098/rstb.2010.0082 20566505 PMC2894961

[ece37156-bib-0053] Naha, D. , Jhala, Y. V. , Qureshi, Q. , Roy, M. , Sankar, K. , & Gopal, R. (2016). Ranging, activity and habitat use by tigers in the mangrove forests of the Sundarban. PLoS One, 11(4), e0152119 10.1371/journal.pone.0152119 27049644 PMC4822765

[ece37156-bib-0054] Nathan, R. , Getz, W. M. , Revilla, E. , Holyoak, M. , Kadmon, R. , Saltz, D. , & Smouse, P. E. (2008). A movement ecology paradigm for unifying organismal movement research. Proceedings of the National Academy of Sciences of the United States of America, 105(49), 19052–19059. 19060196 10.1073/pnas.0800375105 PMC2614714

[ece37156-bib-0055] National Remote Sensing Agency (NRSA) . (2016). Manual on National Landuse/land cover mapping on 1:250,000 using multi‐ temporal IRS P6‐AwiFS data. NRSA, Dept. of Space, Govt. of India, Balanagar, Hyderabad.

[ece37156-bib-0056] Neale, J. C. , & Sacks, B. N. (2001). Food habits and space use of gray foxes in relation to sympatric coyotes and bobcats. Canadian Journal of Zoology, 79(10), 1794–1800. 10.1139/z01-140

[ece37156-bib-0057] Odden, M. , Wegge, P. , & Fredriksen, T. (2010). Do tigers displace leopards? If so, why? Ecological Research, 25, 875–881.

[ece37156-bib-0058] Openstreetmap (2015). Retrieved September 1, 2018, https://www.openstreetmap.org

[ece37156-bib-0059] Papworth, S. K. , Bunnefeld, N. , Slocombe, K. , & Milner‐Gulland, E. J. (2012). Movement ecology of human resource users: Using net squared displacement, biased random bridges and resource utilization functions to quantify hunter and gatherer behaviour. Methods in Ecology Evolution, 3(3), 584–594. 10.1111/j.2041-210X.2012.00189.x

[ece37156-bib-0060] Prange, S. , & Gehrt, S. D. (2007). Response of skunks to a simulated increase in coyote activity. Journal of Mammalogy, 88(4), 1040–1049.

[ece37156-bib-0061] R Core Team (2020). R: A language and environment for statistical computing. R Foundation for Statistical Computing https://www.R‐project.org/

[ece37156-bib-0062] Ripple, W. J. , Estes, J. A. , Beschta, R. L. , Wilmers, C. C. , Ritchie, E. G. , Hebblewhite, M. , Berger, J. , Elmhagen, B. , Letnic, M. , Nelson, M. P. , Schmitz, O. J. , Smith, D. W. , Wallach, A. D. , & Wirsing, A. J. (2014). Status and ecological effects of the world’s largest carnivores. Science, 343(6167), 1241484 10.1126/science.1241484 24408439

[ece37156-bib-0063] Sarkar, M. S. , Ramesh, K. , Johnson, J. A. , Sen, S. , Nigam, P. , Gupta, S. K. , Murthy, R. S. , & Saha, G. K. (2016). Movement and home range characteristics of reintroduced tiger (*Panthera tigris*) population in Panna Tiger Reserve, central India. European Journal of Wildlife Research, 62(5), 537–547. 10.1007/s10344-016-1026-9

[ece37156-bib-0064] Saxena, A. , Chatterjee, N. , Rajvanshi, A. , & Habib, B. (2020). Integrating large mammal behaviour and traffic flow to determine traversability of roads with heterogeneous traffic on a Central Indian Highway. Scientific Reports, 10(1), 18888 10.1038/s41598-020-75810-2 33144654 PMC7642331

[ece37156-bib-0065] Seidensticker, J. , Sunquist, M. E. , & McDougal, C. (1990). Leopards living at the edge of the Royal Chitwan National Park, Nepal In Conservation in developing countries: Problems and prospects (pp. 415–423). Bombay, India: Oxford University Press, Bombay Natural History Society.

[ece37156-bib-0066] Shahi, S. P. (1982). Report of grey wolf (Canis lupus pallipes Sykes) in India‐a preliminary survey. Journal of Bombay Natural History Society, 79, 493–502.

[ece37156-bib-0067] Sharma, D. K. , Maldonado, J. E. , Jhala, Y. V. , & Fleischer, R. C. (2004). Ancient wolf lineages in India. Proceeding of Royal Society of London B‐Biological Science, 271(Suppl. 3), 1–4. 10.1098/rsbl.2003.0071 PMC1809981 15101402

[ece37156-bib-0068] Shrotriya, S. , Lyngdoh, S. , & Habib, B. (2012). Wolves in Trans‐Himalayas: 165 years of taxonomic confusion. Current Science, 103(8), 885–887.

[ece37156-bib-0069] Signer, J. , Fieberg, J. , & Avgar, T. (2019). Animal movement tools (amt): R package for managing tracking data and conducting habitat selection analyses. Ecology and Evolution, 9(2), 880–890. 30766677 10.1002/ece3.4823 PMC6362447

[ece37156-bib-0070] Smith, D. J. L. , Simchareon, S. , Simchareon, A. , Cutter, P. , Gurung, B. , Chundawat, R. S. , McDougal, C. , & Seidensticker, J. (2011). Seasonally dry tropical forest is essential Tiger Habitat In W. J. McShea , S. J. Davies , & N. Bhumpakphan (Eds.), The ecology and conservation of seasonally dry forests in Asia (p. 413). Smithsonian Institution Scholarly Press.

[ece37156-bib-0071] Stevens, F. R. , Gaughan, A. E. , Linard, C. , & Tatem, A. J. (2015). Disaggregating census data for population mapping using random forests with remotely‐sensed and ancillary data. PLoS One, 10(2), e0107042. 25689585 10.1371/journal.pone.0107042 PMC4331277

[ece37156-bib-0072] Sunquist, M. E. (1981). The social organization of tigers (*Panthera tigris*) in Royal Chitawan National park, Nepal. Smithsonian contributions to zoology.

[ece37156-bib-0073] Swingland, I. R. , & Greenwood, P. J. (1983). The ecology of animal movement (pp. 311). Oxford University Press.

[ece37156-bib-0074] Thompson, M. E. , Kahlenberg, S. M. , Gilby, I. C. , & Wrangham, R. W. (2007). Core area quality is associated with variance in reproductive success among female chimpanzees at Kibale National Park. Animal Behaviour, 73(3), 501–512. 10.1016/j.anbehav.2006.09.007

[ece37156-bib-0075] Treves, A. (2009). Hunting for large carnivore conservation. Journal of Applied Ecology, 46(6), 1350–1356. 10.1111/j.1365-2664.2009.01729.x

[ece37156-bib-0076] Trombulak, S. C. , & Frissell, C. A. (2000). Review of ecological effects of roads on terrestrial and aquatic communities. Conservation Biology, 14(1), 18–30. 10.1046/j.1523-1739.2000.99084.x

[ece37156-bib-0077] Tucker, M. A. , Böhning‐Gaese, K. , Fagan, W. F. , Fryxell, J. M. , Van Moorter, B. , Alberts, S. C. , Ali, A. H. , Allen, A. M. , Attias, N. , Avgar, T. , Bartlam‐Brooks, H. , Bayarbaatar, B. , Belant, J. L. , Bertassoni, A. , Beyer, D. , Bidner, L. , van Beest, F. M. , Blake, S. , Blaum, N. , … Mueller, T. (2018). Moving in the Anthropocene: Global reductions in terrestrial mammalian movements. Science, 359(6374), 466–469. 10.1126/science.aam9712 29371471

[ece37156-bib-0078] United Nations, Department of Economic and Social Affairs, Population Division (2017). World Population Prospects: The 2017 Revision, Methodology of the United Nations Population Estimates and Projections. Working Paper No. ESA/P/WP.250. New York: United Nations.

[ece37156-bib-0079] Valeix, M. , Hemson, G. , Loveridge, A. J. , Mills, G. , & Macdonald, D. W. (2012). Behavioural adjustments of a large carnivore to access secondary prey in a human‐dominated landscape. Journal Applied Ecology, 49(1), 73–81. 10.1111/j.1365-2664.2011.02099.x

[ece37156-bib-0080] Van Heerden, J. , Burroughs, R. E. , Dauth, J. , & Dreyer, M. J. (1991). Immobilization of wild dogs (Lycaon pictus) with a tiletamine hydrochloride/zolazepam hydrochloride combination and subsequent evaluation of selected blood chemistry parameters. Journal Wildlife Diseases, 27(2), 225–229. 10.7589/0090-3558-27.2.225 2067044

[ece37156-bib-0081] Wall, J. (2014). Movement Ecology Tools for ArcGIS (ArcMET) vol 10.2.2 vX. www.movementecology.net

[ece37156-bib-0082] Wang, S. W. , & Macdonald, D. W. (2009). Feeding habits and niche partitioning in a predator guild composed of tigers, leopards and dholes in a temperate ecosystem in central Bhutan. Journal of Zoology, 277(4), 275–283.

[ece37156-bib-0083] Webb, S. L. , Dzialak, M. R. , Harju, S. M. , Hayden‐Wing, L. D. , & Winstead, J. B. (2011). Influence of land development on home range use dynamics of female elk. Wildlife Research, 38(2), 163–167. 10.1071/WR10101

[ece37156-bib-0084] Weber, W. , & Rabinowitz, A. (1996). A global perspective on large carnivore conservation. Conservation Biology, 10(4), 1046–1054. 10.1046/j.1523-1739.1996.10041046.x

[ece37156-bib-0085] Wikramanayake, E. D. , Dinerstein, E. , Robinson, J. G. , Karanth, U. , Rabinowitz, A. , Olson, D. , Mathew, T. , Hedao, P. , Conner, M. , Hemley, G. , & Bolze, D. (1998). An ecology‐based method for defining priorities for large mammal conservation: The tiger as case study. Conservation Biology, 12, 865–878. 10.1046/j.1523-1739.1998.96428.x

[ece37156-bib-0086] Woodroffe, R. , & Ginsberg, J. R. (1998). Edge effects and the extinction of populations inside protected areas. Science, 280(5372), 2126–2128. 10.1126/science.280.5372.2126 9641920

[ece37156-bib-0087] World Bank (2015). Agricultural land (% of land area)‐ Open World Bank data. https://data.worldbank.org/indicator/ag.lnd.agri.zs

[ece37156-bib-0088] Zar, J. H. (1984). Biostatistical analysis (2nd ed., pp. 176–179). Prentice‐Hall.

